# Sigma-1 Receptor Modulates CFA-Induced Inflammatory Pain via Sodium Channels in Small DRG Neurons

**DOI:** 10.3390/biom15010073

**Published:** 2025-01-06

**Authors:** Yuanlong Song, Zifen Xu, Liangpin Zhang, Linlin Gao

**Affiliations:** 1 Department of Physiology, School of Basic Medicine, Tongji Medical College, Huazhong University of Science and Technology, 13 Hangkong Rd., Wuhan 430030, China; songyuanlong@hust.edu.cn (Y.S.); xuzifen@wust.edu.cn (Z.X.); zhangliangpin@hust.edu.cn (L.Z.); 2 Hubei Key Laboratory of Drug Target Research and Pharmacodynamic Evaluation, Huazhong University of Science and Technology, 13 Hangkong Rd., Wuhan 430030, China; 3 College of Life Sciences and Health, Wuhan University of Science and Technology, Wuhan 430065, China

**Keywords:** sigma-1 receptor, single-cell PCR, DRG neurons, voltage-gated sodium current, Nav1.9

## Abstract

The sigma-1 receptor (Sig-1R) has emerged as a significant target in the realm of pain management and has been the subject of extensive research. Nonetheless, its specific function in inflammatory pain within dorsal root ganglion (DRG) neurons remains inadequately elucidated. This study utilized whole-cell patch clamp techniques, single-cell real-time PCR, and immunohistochemistry to examine the influence of Sig-1R on inflammatory pain induced by complete Freund’s adjuvant (CFA) in a rat model. Our results revealed several key findings: (1) The expression of Sig-1R was found to be upregulated during the progression of inflammatory pain, with a notable translocation from the cytoplasm to the membrane; (2) Inhibition of peripheral Sig-1R using S1RA resulted in a reduction of CFA-induced allodynia; (3) Activation of Sig-1R through PRE-084 led to a decrease in the fast sodium current in isolated DRG neurons from CFA-treated rats, which was associated with a diminished action potential (AP) peak and maximum depolarizing rate (MDR), as well as an increased rheobase; (4) Furthermore, PRE-084 was observed to enhance the slow component of the sodium current, resulting in hyperpolarization of the threshold potential and an increase in AP firing frequency, alongside an elevation in the mRNA expression of the slow sodium channel Nav1.9 in CFA-treated rats. In conclusion, our findings suggest that the modulation of sodium channels by Sig-1R in DRG neurons plays a significant role in the mechanisms underlying inflammatory pain.

## 1. Introduction

The sigma-1 receptor is a key scaffolding chaperone predominantly enriched in the endoplasmic reticulum membranes associated with mitochondria at the subcellular level [1,2]. In states of cellular stress, Sig-1Rs are activated and translocated to the cell membrane or cytosol to interact with functional proteins [3] and ion channels [4], exerting various biological effects.

Approximately 20% of adults suffer from chronic inflammatory and neuropathic pain. However, existing medications have demonstrated limited effectiveness and are associated with a variety of side effects that significantly restrict their application. Consequently, there is a critical need to discover new pharmacological targets for alleviating pain. Sig-1R represents a highly promising and appealing target for analgesic intervention, given its abundant expression in sensory neurons along the pain transmission pathway, particularly in dorsal root ganglion (DRG) neurons [5,6]. Studies have shown that animals with Sig-1R knockout exhibited reduced allodynia and hyperalgesia during pathological pain states, with no effects on acute pain sensation [7]. Similarly, blocking Sig-1Rs has been effective in decreasing hypersensitivity in pathological pain states, including neuropathic pain induced by various etiologies [8,9,10,11] and inflammatory pain resulting from tissue injury [12]. The Sig-1R blocker S1RA (also known as E-52862) has shown analgesic efficacy in phase II clinical trials [13].

The most intriguing mechanism of pain relief associated with Sig-1R involves its interaction with opioid receptors [12,14,15,16]. This interaction enhances the analgesic potency of opioid drugs while simultaneously reducing their side effects and addictive potential. Additionally, Sig-1R has been shown to modulate chronic pain-related ion channels, such as TRPV1 [17] and TRPA1 [18]. Targeting Sig-1Rs on DRG neurons was a strong and effective strategy for inflammatory pain relief, as blockade of peripheral Sig-1Rs on DRG neurons was sufficient to alleviate inflammatory pain [19,20]; the specific effects of Sig-1R on sodium channel function, particularly in DRG neurons, remain unclear. Sodium channels of various subtypes (Nav1.7 [21], Nav1.8 [22], and Nav1.9 [23]) are extensively expressed in DRG neurons and serve different roles in nociception. Nav1.7 is characterized by fast activation and inactivation, driving the membrane potential toward the threshold and initiating firing. Nav1.8 accelerates the action potential’s rising phase and correlates with the AP peak [24]. In contrast, Nav1.9, which has ultra-slow activation and inactivation kinetics, helps maintain subthreshold repetitive firing [25]. It has been shown that all three of those subtypes of sodium channels contribute to neuropathic and inflammatory pain [26,27,28,29].

In this study, we utilized a CFA-induced inflammatory pain model to examine the effects of Sig-1R on sodium channels. Our findings reveal that activation of Sig-1R has dual effects on the sodium current: it enhances the slow component while inhibiting the fast component. This highlights the potential therapeutic benefits of targeting Sig-1Rs and their interactions with Nav1.9 in pain management.

## 2. Method

### 2.1. The Complete Freund’s Adjuvant (CFA) Treated Animal Model

Juvenile Sprague-Dawley rats, weighing between 150 and 200 g, were accommodated in the animal facility at Tongji Medical College, Huazhong University of Science and Technology, with free access to food and subjected to a natural day/night cycle. To eliminate gender-related differences, only female rats were used. There is no evidence to suggest the existence of sex-dependent differences in pain modulation by the sigma-1 receptor [15]. All experimental procedures were approved by the Animal Welfare Committee of Huazhong University of Science and Technology, and all experiments in this study were conducted in accordance with the guidelines set forth by this committee.

Animals were anesthetized via isoflurane inhalation. To induce inflammation in the entire hind limb, CFA was injected intradermally at the plantar surface of the left hind paw and the lateral region of the left knee (100 μL for each site) [30].

### 2.2. Behavior Test

**Figure 1:**
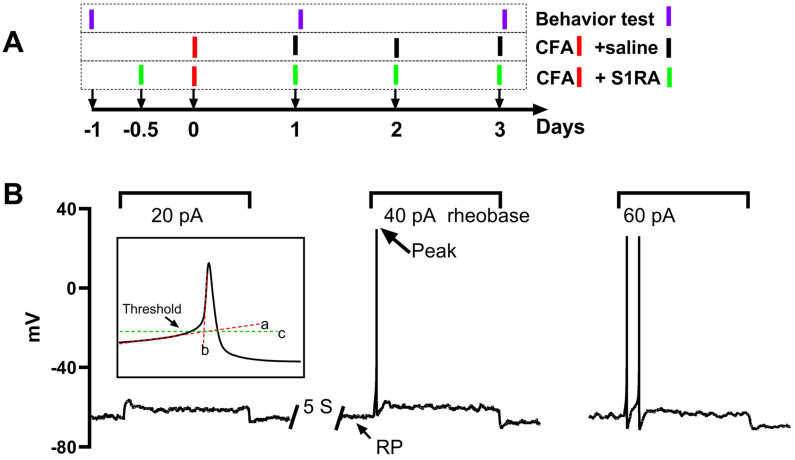
Experimental procedure and protocols for action potential (AP) recording and analysis of AP properties. (**A**): the time scale procedure for CFA injection, S1RA injection, and behavioral tests. (**B**): A series of currents, each lasting two seconds and increased in intensity (20 pA increments), were injected into the DRG neurons repeatedly with a 5 s interval until action potentials were successfully triggered. The minimum current intensity in this example, 40 pA, is denoted as the rheobase. The membrane potential when no current was injected is referred to as the resting potential (RP), and the maximum reached membrane potential during the AP is indicated as the peak (indicated by an arrow). The AP threshold is defined as the critical depolarized membrane potential level at which an AP can be successfully initiated. The threshold level was determined as the intersection point (marked by line c) of the linearly fitted lines of the slow and fast depolarizing phases (lines a and b).

Behaviors were tested on three groups of animals: CFA, S1RA, and sham. The CFA animals received CFA injections and saline injections on three consecutive days. To investigate the role of peripheral Sig-1Rs in the development of CFA-induced inflammatory pain, the selective Sig-1R blocker S1RA was injected (125 μg/20 μL/site) 12 h before CFA administration and daily until the 3rd day after CFA injection. The injection sites were the same as those used for CFA. The sham animal received a saline injection. Behavior tests were performed one day before and one and three days after CFA injection. The test on the 1st and 3rd day after CFA was performed within 2 h of injection (Figure 1A).

All selected CFA animals maintained good health and exhibited indistinguishable levels of exploratory behavior, feeding activity, and weight gain compared to normal rats. For cold and tactile allodynia, 11 and 13 rats were used in each group, respectively.

Mechanical allodynia and cold allodynia were tested as signs of stimulus-evoked pain. To test mechanical allodynia, a series of Von Frey filaments (providing forces of 2, 4, 6, 8, 10, 15, and 26 g. Touch-Test; North Coast Medical, Morgan Hill, CA, USA), from thin to thick, were applied to the plantar surface of each hind paw until withdrawal was achieved, and the force causing withdrawal was recorded as F1. After two minutes, a filament with force one level lower than F1 was applied. This force is recorded as F2. If a withdraw response can be triggered; otherwise, the force of the filament was repeatedly increased one-level higher, with two minutes interval, until a withdraw response was achieved, and the correlated force was recorded as F2. This process was continued until five positive-response forces were determined. The paw withdraw threshold (PWT) was determined as: $\frac{1}{5}\left(\sum_{i=1}^{5}Fi\right)$, where Fi is the ith force.

To test cold allodynia, a drop of acetone was applied using a 1 mL syringe directly to the plantar surface of each hind paw for five consecutive applications. The responses of flinching, licking, or paw-shaking were tested within five seconds of acetone application. The interval between each application was three minutes. For each round of the test, the response was arbitrarily scored according to the present or absence of flinching, licking, or paw shaking, with any of the presence scored as one. The accumulated points of the five rounds were used, with maximum scores of 15.

### 2.3. Investigation of the Electrophysiological Properties of Dissociated DRG Neurons

To avoid possible interruptions of the anesthetics on the electrophysiological properties of DRG neurons [31], animals were euthanized by cervical dislocation. Dorsal root ganglia from L4-6 were dissected and digested at 37 °C in 1 mg/mL trypsin for 30 min, followed by digestion in 1 mg/mL collagenase II for 30–50 min. The enzyme solutions were prepared in DMEM. The dissociated neurons were collected via low-speed centrifugation (1000 rpm for 1 min) and kept in a 4 °C DMEM solution for patch clamp recording within eight hours. The neurons were placed in the perfusion chamber mounted on the stage of an inverted microscope (Zeiss). After attachment, patch clamping was performed at room temperature (25 ± 1 °C). Pipettes with a resistance of 1.5–2.5 MΩ, after being filled with the pipette solution, were used for patch clamp recording. The electrical signals were amplified with Axonpatch-1B (Molecular Devices, CA, USA), digitized with Digidata-1322A (Molecular Devices), and sampled at 50 kHz using the pClamp 8 software suite (Molecular Devices). Data with series resistance higher than 8 MΩ were not used. Images of each patched neuron were taken for offline size measurement. The isolated neurons were dropped into the perfusion chamber, mounted on the stage of the inverted microscope, and allowed five minutes for attachment to the coverslip.

To record action potentials (APs), the neurons were perfused with Artificial Cerebrospinal Fluid (ACSF), which was composed of (in mM): NaCl 124, NaHCO_3_ 26, KCl 3, glucose 10, CaCl_2_ 2, NaH_2_PO_4_ 1.3, and MgCl_2_ 2, and kept saturated with 95% O_2_ and 5% CO_2_. The pipette solution was composed of (in mM): KCl 20, HEPES 10, EGTA 1, Na_2_ATP 2, K^+^ gluconate 120, and MgCl_2_ 2. The pH was adjusted to 7.4 with NaOH. A series of 2 s currents, with increments of 20 pA and intervals of five seconds, were injected to evoke APs under current clamp mode.

The properties of the AP and sodium current were analyzed offline with custom-written Spike2 (version 7.0, Cambridge Electronic Design Limited, Cambridge, UK) scripts. Rheobase was defined as the minimum injected current amplitude required to evoke an AP (Figure 1B). The maximum AP depolarizing rate (MDR) was determined by taking the first derivative of the action potential waveform. The threshold potential was defined as the critical level of the depolarized membrane potential from which the AP was successfully triggered. To determine this threshold, segments of the slow and fast depolarization parts were linearly fitted, respectively (illustrated by lines a and b in the inset of Figure 1B). The crossover point of the two fitting lines indicated the threshold level (illustrated by line c in the inset of Figure 1B).

**Figure 2:**
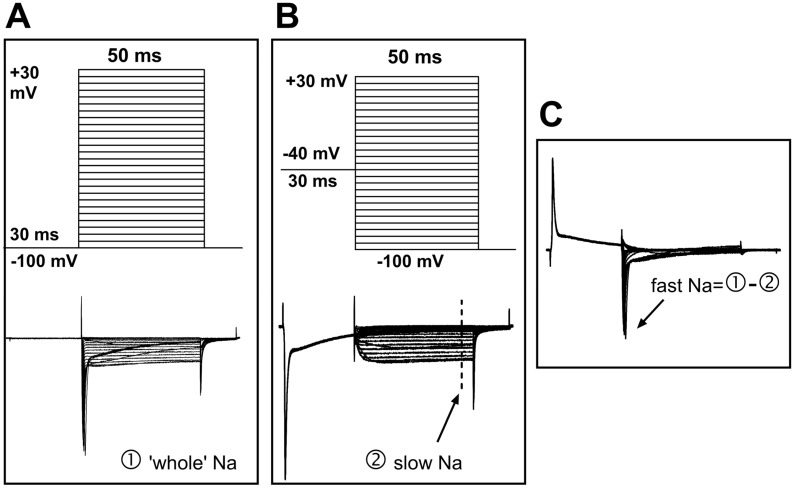
Protocols for the separation of fast and slow sodium components. (**A**) Upper: The command potentials for ‘whole’ sodium current recording. The membrane potential was held at −100 mV for 30 ms before a series of 50 ms potentials (5 mV increments and 2.5 s intervals) were repeatedly applied to the cell. Lower: The ‘whole’ sodium current, which includes both fast and slow components. (**B**) Upper: The command potentials for slow sodium current recording. The fast component was deactivated with a depolarizing pre-pulse (−40 mV), which produced the slow sodium current (lower). The current amplitude was measured at steady state (marked by the dashed line). (**C**) Offline subtraction was performed to generate the fast sodium current, which was measured at the peak level (indicated by an arrow).

To record sodium current, the NaCl in the ACSF was replaced with equal molar TEA-Cl, and the pipette solution was composed of (in mM) CsCl 125, NaCl 13, TEACl 20, HEPES 5, Na_2_ATP 5, and EGTA 10. This change reduced the external/internal sodium gradient and thus controlled the sodium current amplitude at a relatively low level; this was helpful in reducing the voltage error generated by series resistance, which could otherwise result from very high sodium current amplitudes if physiological solutions were used. To separate the fast and slow sodium current components, two sets of command potentials were applied under voltage clamp mode sequentially. The potential of the target cell was held at −100 mV for 30 ms and then depolarized from −110 mV to +40 mV for 50 ms (with a step of +5 mV and an interval of 2.5 s) (Figure 2A, upper). This activated the whole sodium current (both fast and slow components included; Figure 2A, bottom). The membrane potential of the cell was then set to −40 mV to deactivate the fast sodium component before the potential was depolarized from −100 mV to +40 mV (Figure 2B, upper). Thus, the slow component of the sodium current was separated (Figure 2B, bottom). The fast sodium component was obtained via offline subtraction of the slow from the whole sodium current (Figure 2C).

### 2.4. Immunohistochemistry

Rats were euthanized with an overdose of sodium pentobarbital (i.p. 60 mg/kg) and were perfused transcardially with saline solution, followed by 4% paraformaldehyde in 0.1 M phosphate buffer (pH 7.4). The bilateral L4 and L5 dorsal root ganglia (DRGs) were removed, post-fixed in 4% paraformaldehyde for 1 h, and cryoprotected in 30% sucrose in 0.1 M phosphate buffer for 12 h. The DRGs were embedded in OCT embedding medium and stored at −80°C until processing. The DRGs were sectioned at 7 μm thickness using a cryostat and thaw-mounted onto electrostatic glass slides. Sections were washed in phosphate-buffered saline (PBS) containing 0.2% Triton X-100 three times for five minutes, blocked in SuperBlock solution (5% bovine serum albumin, 10% fetal bovine serum, 0.2% Triton X-100 in PBS) for two hours at room temperature, and then incubated overnight at 4 °C with Sig1-R primary antibody diluted in blocking solution. Sections were washed three times with PBS and incubated for four hours in secondary antibody (Alexa Fluor 555 goat anti-rabbit, 1:1000 dilution, Invitrogen, Shanghai, China). Hoechst (1 µg/mL) was added to the secondary antibody solution for nuclear staining. Sections were washed three times in PBS prior to coverslipping with Fluoroshield (F6182-20mL, Sigma, Shanghai, China). Images were acquired using a Leica TCS SPE confocal microscope (NJ, USA) with the Leica Application Suite.

**Figure 3:**
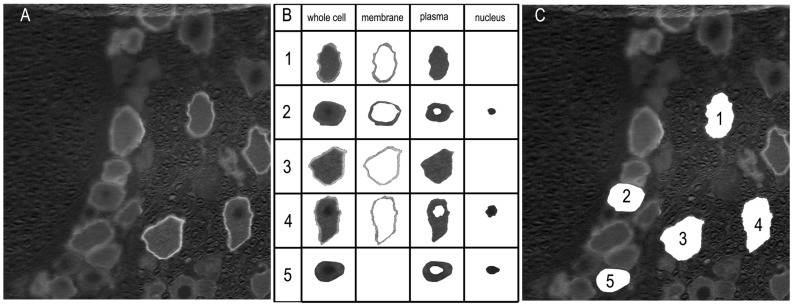
Labeling analysis of the immunofluorescent images. (**A**) The images were converted to grayscale. The labeling intensity was indicated by arbitrary pixel intensity (from 0 to 255). (**B**) Boundaries of the whole cell, membrane, plasma, and nucleus regions were determined automatically using a custom-designed application. The accuracy of the segmentation was verified by the user, and corrections were made if necessary. The example cells 1, 3, and 4 were automatically determined, while the isolations of cells 2 and 5 resulted from manual intervention. (**C**) Locations of the extracted neurons are displayed.

The labeling of Sig-1R was analyzed with a custom-designed application based on the MATLAB App Designer framework (MATLAB Version: 24.1.0.2628055 (R2024a) Update 4). The RGB images were converted to grayscale format (Figure 3A). An interactive segmentation method was applied to delineate the whole-cell boundary and, if labeled, the membrane and nucleus regions (Figure 3B). These regions were first automatically determined, and for each cell, the user was prompted to confirm their accuracy. Manual adjustments were made as necessary (Figure 3B, cells 2 and 5). The number of pixels in each region was counted, and the mean pixel intensity was calculated. The whole cell area was defined as the enclosed pixel number multiplied by the area of one pixel, which was determined according to the scale bar. The diameter of the neuron was calculated using the equation: Diameter = $2\times \sqrt{\frac{Area}{\pi }}$.

### 2.5. Single-Cell PCR

To reduce the interference from cell type heterogeneity, real-time PCR was performed at the single-cell level. The detailed method has been described previously [32]. In brief, DRG neurons with diameters less than 30 μm were collected using a modified patch-clamp pipette. To evaluate the effect of the Sig1-R agonist PRE-084, DRG neurons were collected from six sham rats and six rats after three days of CFA injection (CFA3D); thus, a total of 12 groups (six sham + six CFA3D) of neurons were obtained. These 12 groups of neurons were thoroughly mixed and then halved isovolumetrically. One portion was used as control, and the other was treated with PRE-084 (10 μM) for 30 min before further processing. Due to this grouping method, we regard the objects before and after the PRE-084 treatment as ‘identical’; thus, a paired *t*-test was applied to evaluate the effect of PRE-084. mRNA was extracted using the Dynabeads mRNA DIRECT Micro Kit, and the mRNA was reverse transcribed using the Superscript VILO cDNA Synthesis Kit.

Primers for *Nav1.6–1.9* were designed to span the exon/exon boundaries using Primer Express 2.0 (Applied Biosystems, Thermo Fisher Scientific, MA, USA). This design avoids co-amplification of any contaminating genomic DNA, which could compromise the specificity of the amplification. The specificity of the primers was verified using BLAST (http://www.ncbi.nlm.nih.gov/BLAST (accessed on 1 December 2014)). The primers were ordered from Sangon (Shanghai, China), and their sequences are listed in Table 1.

A 10 μL reaction system was prepared and run in a PCR machine (CFX Connect Real-time system, Bio-Rad Laboratories, Inc., Shanghai, China). The reaction system consisted of 5 μL of 2X reaction buffer (Power SYBR Green PCR Master Mix, Applied Biosystems, Shanghai, China, catalog no.: 4368708), 1 μL of 10× primer mix, template cDNA, and RNAase-free water (Takara, Beijing, China). The concentration of the primer mix was 2 µmol/L, resulting in an optimized final concentration of 200 nmol/L. GAPDH was used as the housekeeping gene. The quantification method was a modification of the 2^−ΔΔCT^ method; instead of assuming an amplification efficiency of 2, the amplification efficiency for each transcript in this experiment was estimated using the sigmoidal curve-fitting method described by Liu et al. [33].

### 2.6. Chemicals

The following chemicals were used: CFA (Sigma, St. Louis, MO, USA), Sig1-R primary antibody (Rabbit polyclonal antibody, 1:2000, A5479, ABclonal, Wuhan, China), PRE-084 (HY-18100A, MedChemExpress, Shanghai, China), S1RA (HY-18099, MedChemExpress, China), PCR kits (Invitrogen, Shanghai, China; Dynabeads mRNA DIRECT Micro Kit, catalog no.: 610.21; Superscript VILO cDNA Synthesis Kit, catalog no.: 11754-50), Collagenase II (Worthington Biochemical Corp., NJ, USA, S4B70644176), and Trypsin (Sigma, Shanghai, China, T-8003). CFA (Sigma, St. Louis, MO, USA) was suspended in an oil/saline emulsion (1:1). One ml of CFA contains 1 mg of heat-killed and dried mycobacterium tuberculosis (strain H37Ra, ATCC25177), 0.85 mL of paraffin oil, and 0.15 mL of mannide monooleate. All other chemicals not stated were obtained from Sigma.

### 2.7. Analysis

Statistical analysis was performed using SPSS 27 (IBM, New York, NY, USA). A paired or independent *t*-test was used to compare differences within or between groups if the data were normally distributed; otherwise, the Wilcoxon matched-pairs test or Mann–Whitney test was applied for rank comparisons of the paired or unpaired data, respectively. The Pearson’s Chi-Square analysis was performed to compare the percentage of neurons with membrane or nucleus-region labeling. Figures were plotted using GraphPad Prism 9.0 (GraphPad Software, Boston, MA, USA).

## 3. Results

### 3.1. CFA Treatment Increased the Expression of Sig-1R and Led to Its Recruitment from Intracellular Compartments Toward the Membrane

In this study, labeling information for 679 neurons was collected, with diameters ranging from 17 to 104.5 μm and a mean diameter of 47.3 μm. The values for the 10th, 30th, and 50th percentiles were 31.3, 38.9, and 45.1 μm, respectively (Supplementary Table S1, Supplementary Figure S1).

For the special interest of the current study, properties of small neurons, with a diameter ≤ 30 μm, were analyzed. Small neurons are the major types responsible for nociception [33].

**Figure 4:**
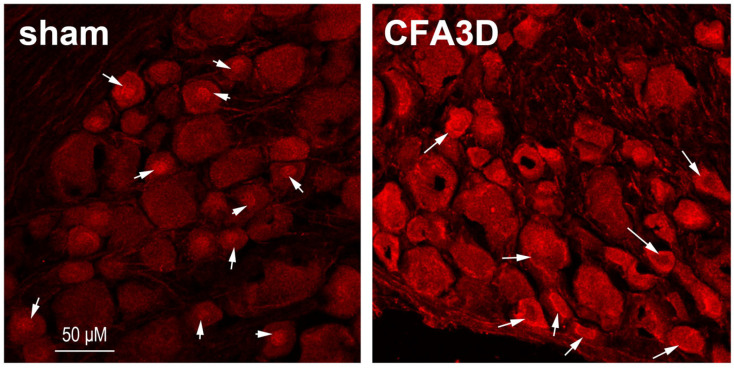
Immunofluorescent labeling of Sig-1R from sham and rats after three days of CFA injection. In the sham animals, a high proportion of Sig-1Rs aggregated in the central nuclear regions (indicated by an arrow). After three days of CFA injection (CFA3D), Sig-1R trafficking occurred toward the membranous region (indicated by an arrow). Compared with sham, the labeling intensity of Sig-1R in CFA3D increased significantly.

CFA caused membrane ward trafficking of Sig-1R. In sham animals, Sig-1R was aggregated in the central nucleus region in 53.6% of neurons (15 out of 28), which was reduced to 26.7 after CFA3D (12 out of 48) (*p* < 0.05, Pear Chi-Square test). Consistently, the membrane labeling ratio was increased from 0% (sham) to 28.9% (CFA3D, 13 out of 48) (*p* < 0.01, Pearson’s Chi-Square test). As illustrated in Figure 4, prominent cytoplasmic-to-membrane regional transition of Sig-1R was observed after CFA3D.

CFA increased the Sig-1R labeling intensity, which was enhanced from 95.6 ± 3.5 (sham, n = 28) to 126.5 ± 4.4 (CFA3D, n = 45) (*p* < 0.01, independent *t*-test).

### 3.2. Blocking Sig-1R Partially Rescued CFA-Induced Allodynia

To investigate the effect of the enhanced membrane-trafficked Sig-1R that occurred after CFA3D, the Sig-1R blocker S1RA was administered, and its effects on animal behaviors were examined.

**Figure 5:**
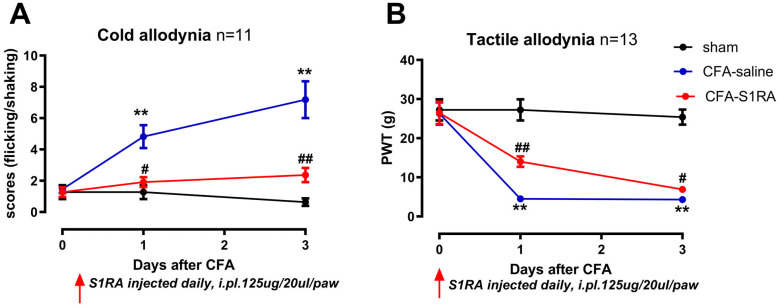
The effects of CFA injection and S1RA on cold and tactile allodynia. (**A**) CFA injection increased the cold allodynia scores, which were decreased by S1RA administration. (**B**) CFA injection decreased the paw withdrawal thresholds (PWTs) for tactile allodynia, which were increased by S1RA administration. CFA-saline: CFA injection followed with three daily saline injections; CFA-S1RA: S1RA injected 12 h before CFA and daily injection until the 3rd day. Injections were administered intradermally at the plantar surface of the left hind paw and the lateral region of the left knee. ( ** *p* < 0.01. CFA vs. sham; # *p* < 0.05, ## *p* < 0.01, S1RA vs. CFA, Mann–Whitney test).

Compared with sham, CFA injection induced prominent mechanical and cold allodynia (at day one and three, Figure 5). For instance, three days after CFA injection, the mechanical paw withdrawal threshold was significantly reduced from 26.0 g to 4.0 g (Figure 5B), and the scores of cold allodynia increased from 0 to 7 (Figure 5A) (sham vs. CFA, Mann–Whitney test, *p* < 0.01).

Administration of the Sig-1R antagonist S1RA partially rescued CFA-induced allodynia. Scores of cold allodynia after CFA at day three were significantly decreased from 7 to 2, and the PWT increased from 4.0 g to 6.0 g (CFA vs. S1RA, Mann–Whitney test, *p* < 0.05).

### 3.3. Effects of Sig1-R Agonist on the Electrophysiological Properties of DRG Neurons

The AP data were collected from 10 sham and 10 CFA3D neurons, from three and four animals, respectively.

**Figure 6:**
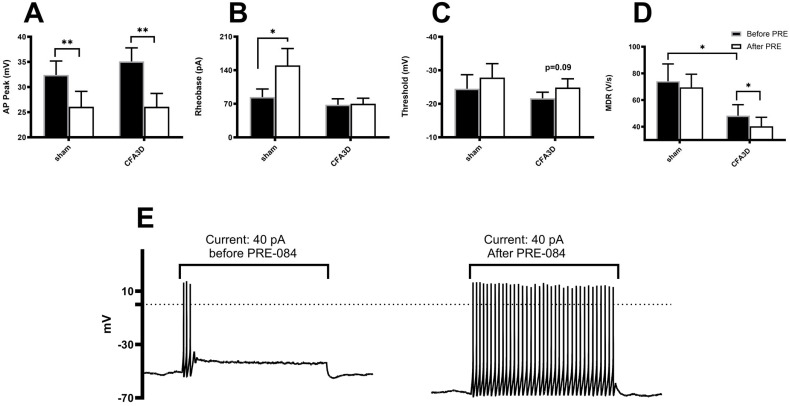
The effects of the Sig-1R agonist, PRE-084, on the electrophysiological properties of small DRG neurons (≤30 μm). (**A**) PRE-084 significantly reduced the peak amplitude of the action potentials (APs) in both sham and three days after CFA injection (CFA3D) animals (*p* < 0.01, paired *t*-test). (**B**) PRE-084 decreased the rheobase in sham animals but had no effect in CFA3D animals. (**C**) PRE-084 showed a trend toward hyperpolarizing the AP firing threshold potential (*p* = 0.09). (**D**) PRE-084 significantly increased the maximum depolarization rate (MDR) of the AP in CFA3D animals. (*p* < 0.05, paired *t*-test). Compared with sham, CFA3D decreased the MDR (*p* < 0.05, independent *t*-test). (**E**) PRE-084 increased the firing frequency in CFA3D neurons. *: *p* < 0.05; **: *p* < 0.01.

Compared with sham, CFA3D significantly reduced the AP maximal depolarizing rate (MDR) from 74.1 ± 12.9 to 48.3 ± 8.2 V/s (sham vs. CFA3D, *p* < 0.05, independent *t*-test; Figure 6D); CFA3D did not alter the action potential (AP) properties of peak, rheobase, and threshold potential (Figure 6A–C).

The Sig-1R agonist PRE-084 reduced the AP peak in both sham and CFA3D animals. Specifically, in CFA3D, the AP peak was decreased from 35.1 ± 2.8 to 26.1 ± 2.6 mV (before vs. after PRE-084, *p* < 0.01, paired *t*-test. Figure 6A).

PRE-084 significantly increased the rheobase in the sham group (from 84 ± 16.8 to 150 ± 35.3 pA); however, this effect was not observed in the CFA3D group (Figure 6B).

In CFA3D, PRE-084 showed a trend toward hyperpolarizing the threshold, although this effect did not reach statistical significance (*p* = 0.09, Figure 6C).

In CFA3D, PRE-084 decreased the MDR from 48.3 ± 8.2 to 40.3 ± 6.8 V/s (before vs. after PRE-084, *p* < 0.05, paired *t*-test; Figure 6D); this effect was not observed in the sham animals.

PRE-084 significantly increased the AP firing frequency, recorded at rheobase (Figure 6E).

### 3.4. Effects of Sig1-R Agonist on Fast and Slow Sodium Current

Sodium current was recorded from 17 neurons of four sham and 12 neurons of three CFA3D animals. Activating the Sig-1R in isolated DRG neurons showed opposite effects on the fast and slow sodium components. Sig-1R activation generally inhibited the sodium fast component but stimulated the slow components.

**Figure 7:**
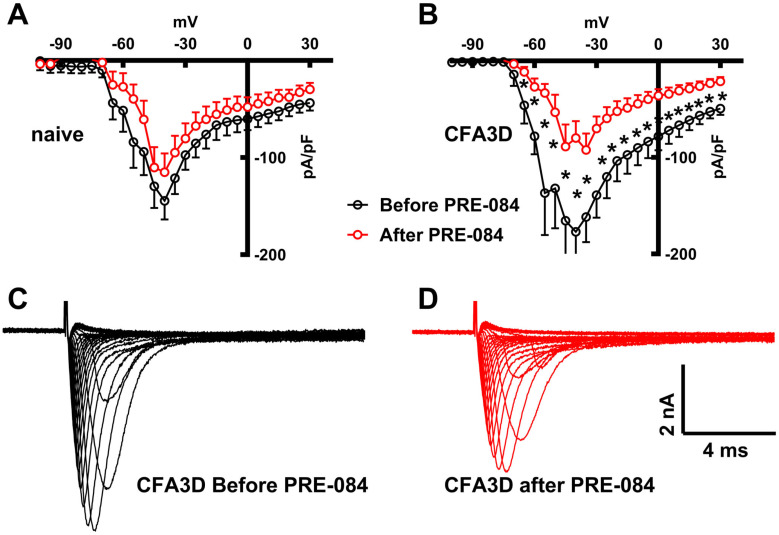
The effects of PRE-084 on the fast components of sodium current. (**A**,**B**) The inhibitory effect of PRE-084 on the fast sodium current in sham (**A**) and three days after CFA injection (CFA3D) animals (**B**). The inhibition was statistically significant only in CFA3D animals (* *p* < 0.05, before vs. after PRE-084, paired *t*-test). (**C**,**D**) Example sodium currents recorded from a CFA3D neuron. The application of PRE-084 inhibited this current (before (**C**) vs. after (**D**)).

The fast sodium current components were reduced significantly by PRE-084 in CFA3D at test potentials from −65 mV to +30 mV (*p* < 0.05, paired *t*-test, marked by asterisks in Figure 7B), as demonstrated by Figure 7C (before) and Figure 7D (after PRE-084). Although a similar inhibitory pattern was observed in sham, the differences did not reach the level of statistical significance (Figure 7A).

**Figure 8:**
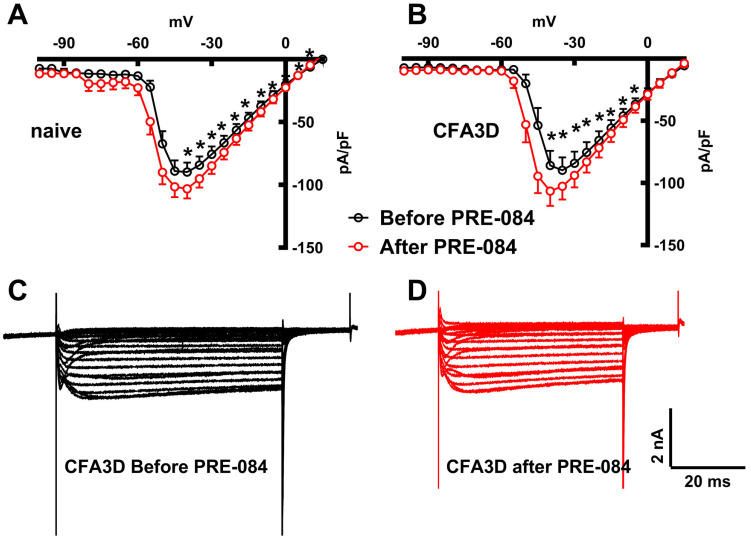
The effects of PRE-084 on the slow components of sodium current. (**A**,**B**) The enhancing effect of PRE-084 on the slow sodium current in sham (**A**) and three days after CFA injection (CFA3D) animals (**B**) (* *p* < 0.05, before vs. after PRE-084, paired *t*-test). (**C**,**D**) Example sodium currents recorded from a CFA3D neuron. The application of PRE-084 increased this current (before (**C**) vs. after (**D**)).

In contrast to its effect on the fast sodium current, PRE-084 enhanced the slow component of the sodium current. This effect was observed in both the sham (from −45 to +20 mV) and CFA3D (from −40 to −5 mV) groups (Figure 8A,B) (*p* < 0.05, paired *t*-test). Figure 8C,D illustrate the example slow sodium current traces before and after the administration of PRE-084.

### 3.5. Effects of Sig-1R Agonist on the mRNA Levels of Nav1.6-1.9

**Figure 9:**
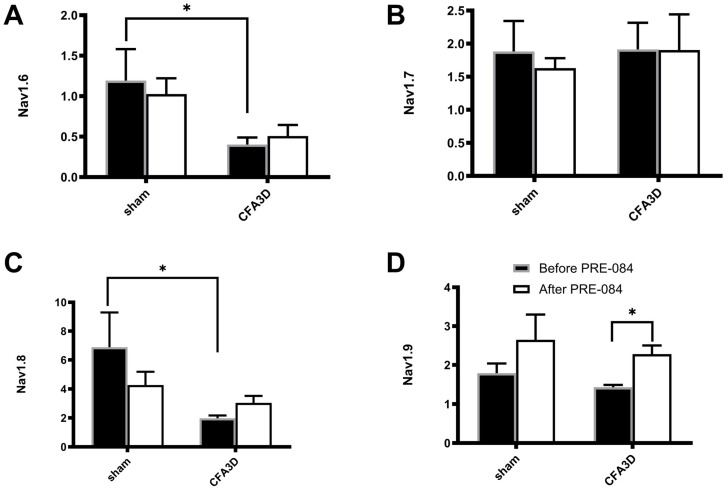
The effects of PRE-084 on the mRNA abundance of *Nav1.6-1.9*. Three days after CFA injection (CFA3D) reduced the relative mRNA abundance of *Nav1.6* (**A**) and *Nav1.8* (**C**) (* *p* < 0.05, sham vs. CFA3D, independent *t*-test). PRE-084 increased the relative mRNA abundance of *Nav1.9* (**D**) (* *p* < 0.05, before vs. after PRE-084, paired *t*-test) and had no effect on *Nav1.6* or *Nav1.8* (**A**–**C**).

Of the four tested mRNA sodium channel subtypes, *Nav1.6-1.9*, CFA 3D decreased the mRNA level of *Nav1.6* and *Nav1.8*, while the level of *Nav1.7* and *Nav1.9* was not altered. Specifically, the relative mRNA abundance of Nav1.6 was reduced from 1.19 ± 0.39 to 0.40 ± 0.09 (Figure 9A) and that of *Nav1.8*, reduced from 6.89 ± 2.4 to 1.98 ± 0.20 (Figure 9C) (sham vs. CFA3D, *p* < 0.05, independent *t*-test).

PRE-084 enhanced the expression level of *Nav1.9* (Figure 9D) but had no effects on *Nav1.6-1.7* (Figure 9A–C). The promoting effect on *Nav1.9* was statistically significant in CFA3D (from 1.43 ± 0.06 to 2.28 ± 0.22, *p* < 0.05).

## 4. Discussion

The current study has made several key discoveries that shed light on the role of peripheral Sig-1Rs in modulating inflammatory pain. For the special interest of the current, properties of small neurons, with a diameter ≤ 30 μm, were analyzed. Firstly, we found that Sig-1R immunofluorescent staining was significantly upregulated in small DRG neurons three days after CFA injection, with a shift of its localization from the nucleus to the cytoplasm and cell membrane. Secondly, local administration of Sig-1R antagonists effectively reversed the CFA-induced mechanical and cold allodynia, highlighting the therapeutic potential of peripheral Sig-1Rs in pain management. Furthermore, in CFA-treated small DRG neurons, Sig-1R activation reduced fast-inactivating sodium currents and increased slow-inactivating sodium currents; consequently, Sig-1R activation reduced the height of action potentials (Figure 6A) and the depolarizing rate (Figure 6D), while enhancing the evoked firing frequency. Lastly, the single-cell real-time PCR analysis revealed that activation of Sig-1R significantly enhanced the expression of Nav1.9 mRNA in small DRG neurons following CFA.

In the CFA-induced inflammatory pain model, a prominent trafficking of Sig-1R from the nucleus to the membrane region was observed. This suggests the potential importance of Sig-1R in modulating inflammatory pain, supported by substantial evidence [15]. Consistently, chronic application of the Sig-1R antagonist S1RA, in this study, efficiently reduced cold allodynia and mechanical allodynia (Figure 5). Similar behavioral responses were observed, confirming the involvement of Sig-1R in pain management [20,34].

The observed toward-membrane trafficking is an essential property of Sig-1R under stress conditions [35,36]. In the current study, Sig-1R was aggregated in the nucleus region of DRG neurons in sham animals (Figure 4). This is not consistent with the mainstream findings that Sig-1R is mainly distributed around the endoplasmic reticulum (ER) [37]. Miki et al. however, confirmed that Sig-1R can be sequestered in the nucleus, and shuttling between the nucleus and cytoplasm occurs in various neurodegenerative diseases [38]. Several factors may contribute to this discrepancy, including species differences, immunohistochemistry processing procedures, and particularly the quality and specificity of antibodies used [6].

At the peripheral level, altered electrical excitability of neurons is an important mechanism for pain sensation. Voltage-gated sodium channels are critically important for electrogenesis and nerve impulse conduction [39]. The Sig-1R can modulate sodium channels. For example, there is evidence showing that Sig-1R can directly interact with the cardiac sodium subtype Nav1.5 and inhibit this channel [40]. Given the close link between Sig-1R and inflammatory pain, two questions arise: (1) Does Sig-1R activation alter the electrical properties of DRG neurons? (2) How do sodium currents interact with this process? Or whether and how are sodium currents getting involved in those processes?

The upregulation and membrane translocation of Sig-1R after CFA3D (Figure 4), along with the effectiveness of the Sig-1R blocker in alleviating allodynia (Figure 5), suggest an overactivated status of this protein during inflammatory pain. However, in isolated DRG neurons, administration of a Sig-1R agonist, PRE-084, led to unexpected results; instead of increasing neuronal excitability, PRE-084 significantly reduced the amplitude of the action potential (AP), decreased the depolarizing rate of the AP (in both sham and CFA3D), and increased the rheobase. These findings indicate that APs are more difficult to initiate and, once triggered, are difficult to conduct. This paradoxical effect of activating Sig-1R on AP contrasts with the dampening effect on inflammatory pain observed after blocking Sig-1R.

It is generally accepted that the fast sodium current, characterized by rapid activation and inactivation, is the major current influx during the upstroke phase of the AP. This current directly correlates with the depolarizing rate and amplitude of the AP and consequently affects neuronal excitability and impulse conductivity. The fast sodium current is sensitive to tetrodotoxin (TTX) and is mediated by the sodium channel subtypes Nav1.6 and Nav1.7 in peripheral sensory neurons, with Nav1.6 being the predominant form [41]. Although the strong inhibitory effects of the Sig-1R agonist on AP properties suggest inhibition of the fast sodium components, there is no information on how Sig-1R interacts with the fast sodium current in DRG neurons. A major challenge is separating the fast from the other sodium components. Of the nine identified sodium channel subtypes (Nav1.1-1.9), all except Nav1.4 are detectable in the peripheral sensory system [41]. In addition to the TTX-sensitive fast sodium component, the TTX-insensitive slow sodium component is typically mixed in the recorded current using conventional patch clamp techniques. The commonly used chemical blocking methods are unsuitable in this case, as a specific blocker for Nav1.9 is not available. Nav1.9 is believed to be the major sodium channel subtype underlying the slow sodium component [25].

In this experiment, a voltage inactivation strategy was employed to inactivate the fast TTX-sensitive component, allowing for the separation of both the TTX-sensitive fast component and the TTX-insensitive sodium components. This strategy relies on the essential property of the TTX-sensitive sodium current, where its deactivation depends on thorough repolarization. Thus, a pre-depolarized potential (Figure 2) eliminates the fast components from the recorded currents.

Consistent with its inhibitory effect on the AP component, the Sig-1R agonist PRE-084 inhibited the fast sodium current (Figure 7). This is the first report, to our knowledge, of Sig-1R activation affecting the fast sodium current on DRG neurons. To understand the mechanism behind this phenomenon, we investigated the effect of PRE-084 on the expression of the major fast sodium subtypes Nav1.6 and Nav1.7 at the transcriptional level. No significant changes, however, were observed before and after PRE-084 treatment, suggesting that this modulation is unlikely to occur at the transcriptional level. The work of Dilshan et al. in transfected cell lines, using atomic force microscopy imaging, confirmed direct binding of Sig-1R to the Nav1.5 channel with 4-fold symmetry. This binding is crucial for the gating of the channel, as knocking down Sig-1R in co-expressed cell lines reduced the Nav1.5-mediated current [40]. Together with the fact that the Sig-1R ligands haloperidol and (+)-pentazocine disrupted this binding [40], along with the high conservation of sodium channel subtypes [42], it is reasonable to deduce that the inhibitory effect of PRE-084 on the fast sodium component is a consequence of its interruption of the Sig-1R-sodium channel interaction. Other possible mechanisms include direct ligand inhibition of the channels, as reported for Nav1.2 and Nav1.4 [43].

Although the inhibitory effect of Sig-1R activation on the fast sodium current and the correlated AP properties quenched our initial hypothesis that the fast sodium channel might mediate pain alleviation via Sig-1R, the enhancing effect of Sig-1R activation on the slow sodium component makes it a promising target.

In contrast to its inhibitory effect on the fast sodium current, the opposite effect of PRE-084 on the slow sodium current (Figure 8) may be a transcriptional-level result. qPCR quantification confirmed that the expression level of Nav1.9, but not Nav1.8, was significantly enhanced by PRE-084 treatment. Consistent with this, the slow sodium current observed in this experiment was likely mediated by Nav1.9 rather than Nav1.8. This assertion is based on the high similarity of the current dynamics with that of Nav1.9 currents recorded from transfected systems, characterized with very slow inactivation (producing a persistent current), a property that is distinctly different from that mediated by Nav1.8, which activates and inactivates more rapidly [25,41]. Due to its slow inactivation, we hypothesize that the sustained inward current, following activation of the slow sodium channel, may promote increased repetitive firing of neurons as observed in this study (Figure 6E). Further experiments are needed to validate this hypothesis.

In an effort to understand the mechanism of Sig-1R activation on AP properties, the effect of the Sig-1R antagonist was also evaluated. Following the administration of PRE-084 (the agonist), the antagonist S1RA was applied for approximately 20 min. The effect of S1RA on AP properties (Supplementary Figure S2) was similar to that of PRE-084 (Figure 7). In other words, the inhibitory effect of PRE-084 could not be reversed by S1RA. Several hypotheses can explain this observation. Firstly, Sig-1R may not function as a conventional ’receptor’, as ligand binding generally does not activate downstream signaling pathways [37]. Thus, the antagonist might not reverse the effects of the agonist as traditionally understood. Secondly, due to the complexity of the receptor, PRE-084 and S1RA may not compete for the same binding site. Thirdly, the agonist and antagonist may exert their effects via different pathways; it has been confirmed that the Sig-1R agonist (+)-SKF 10,047 can inhibit Nav1.2/1.4, while the Sig-1R antagonist does not block this inhibitory effect [42]. Finally, it is possible that both ligands, whether agonist or antagonist, modulate the sodium channel in the same direction under this specific circumstance. For example, haloperidol and (+)-pentazocine are regarded as antagonists and agonists of Sig-1R, respectively [44]; both exert the same effect of disrupting the Sig-1R-Nav1.5 binding [40].

Special notes about this article: In this study, only small-sized DRG neurons were included because these small-sized neurons correspond to nociception [45] and the Nav1.9-mediated slow sodium current presented uniquely in this group of neurons [41].

In conclusion, our study illustrates that the upregulation, translocation, and activation of Sig-1Rs not only resulted in an immediate increase in the slow inactivation current but also significantly enhanced the expression of *Nav1.9* mRNA. This investigation provides new insights into the role of peripheral Sig-1Rs in the modulation of sodium channels, particularly Nav1.9, in inflammatory pain and underscores the potential therapeutic implications of targeting peripheral Sig-1Rs and their interactions with sodium channels for pain management.

## Figures and Tables

**Table 1:** The sequence of the primers.

Primers	Forward (5’-3’)	Reverse (5’-3’)
*Nav1.6*	GAAGAGCTGGAAGAGTCTCAGAGAAA	AAACTTATACCAGCACGGTGGG
*Nav1.7*	GAGCCCGTAAACGCAGATGA	CACACAACCATCTGTAAAGCAGG
*Nav1.8*	GGCTGGATGGACATAATGTATGC	ACTGTTGATCTCTCCGGAATCAA
*Nav1.9*	GACGATGCCTCTAAAAATCCACA	GGACAGTCGTTTGGTCTGCTC

## Data Availability

The original contributions presented in this study are included in the article/Supplementary Material. Further inquiries can be directed to the corresponding author(s).

## Supplementary Materials

The following supporting information can be downloaded at: https://www.mdpi.com/article/10.3390/biom15010073/s1, Figure S1: Neuron diameter histogram; Figure S2: The effects of the Sig-1R agonist, S1RA, on the electrophysiological properties of small DRG neurons in sham and CFA3D animals; Table S1: Frequencies of neurons with different diameters.
