# Supplementary material for: Sigma-1 Receptor Modulates CFA-Induced Inflammatory Pain via Sodium Channels in Small DRG Neurons
Source: Biomolecules. 2025 Jan 6;15(1):73. doi: 10.3390/biom15010073 (PMC11764217; doi:10.3390/biom15010073)
Supplement: Supplementary file 1 [file biomolecules-15-00073-s001.zip › biomolecules-3402604-supplementary.pdf]

Supplement Table S1. Frequencies of neurons with different diameters

N	Valid	679
	Missing	0
Mean		47.3
Std. Deviation		14.1
Minimum		17.0
Maximum		104.5
Percentiles	10	31.3
	20	35.9
	30	38.9
	40	42.0
	50	45.1
	60	48.5
	70	52.6
	80	57.8
	90	66.7

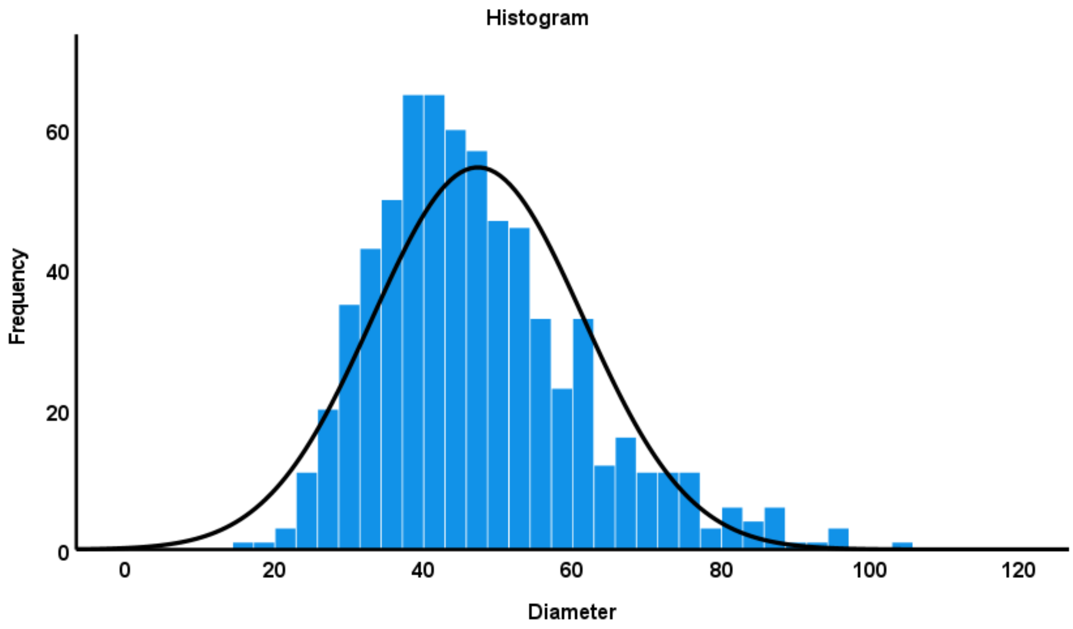

Supplement Figure S1. Neuron diameter histogram.
The histogram of the diameter of the DRG neurons extracted from the immuno- labelled images.

Supplementary Figure S2

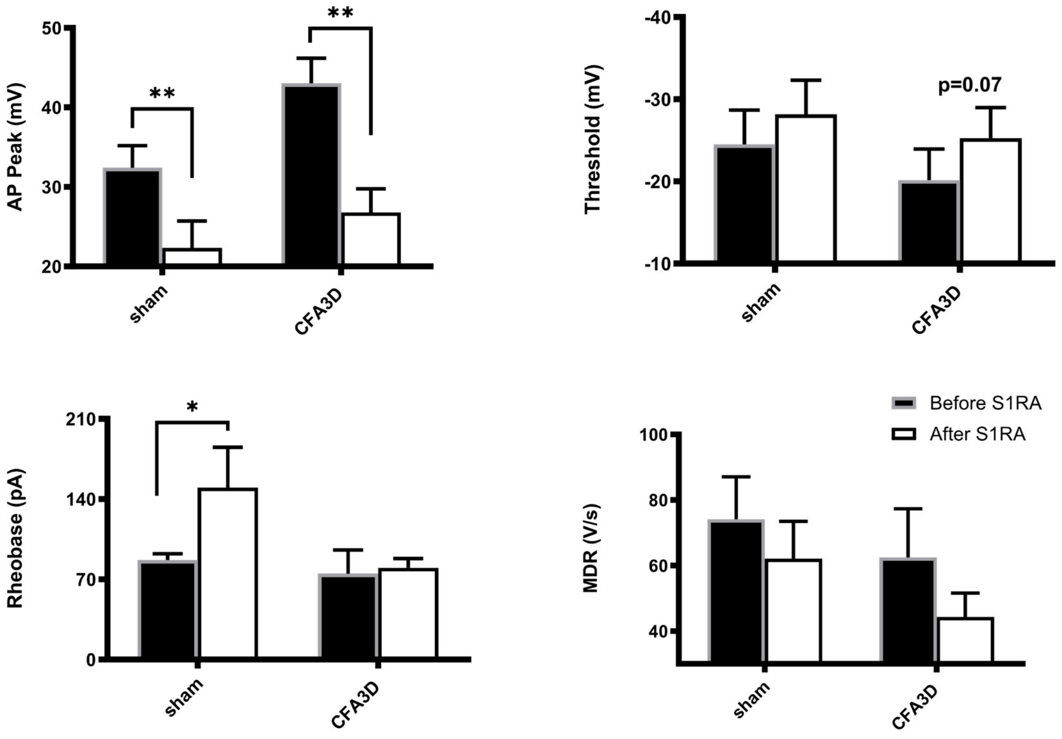

The effects of the Sig-1R agonist, S1RA, on the electrophysiological properties of small DRG neurons in sham and CFA3D animals.

S1RA significantly reduced the peak amplitude of the action potentials (APs) in both sham and CFA3D animals ($p<0.01$, paired t test). S1RA decreased the rheobase in sham animals but had no effect in CFA3D animals.
